# Supplementary material for: Climate and land management accelerate the Brazilian water cycle
Source: Nat Commun. 2022 Sep 1;13:5136. doi: 10.1038/s41467-022-32580-x (PMC9436950; doi:10.1038/s41467-022-32580-x)
Supplement: Supplementary file 1 — Supplementary Information [file 41467_2022_32580_MOESM1_ESM.pdf]

Supplementary Information for

Climate and land management accelerate the Brazilian water cycle

Vinícius B. P. Chagas[1*], Pedro L. B. Chaffe[2*], Günter Blöschl[3].

[1] Graduate Program of Environmental Engineering, Federal University of Santa Catarina, Florianopolis, Brazil.
[2] Department of Sanitary and Environmental Engineering, Federal University of Santa Catarina, Florianopolis, Brazil.
[3] Institute of Hydraulic Engineering and Water Resources Management, Technische Universität Wien, Vienna, Austria.

* Corresponding authors. vbchagas@gmail.com and pedro.chaffe@ufsc.br

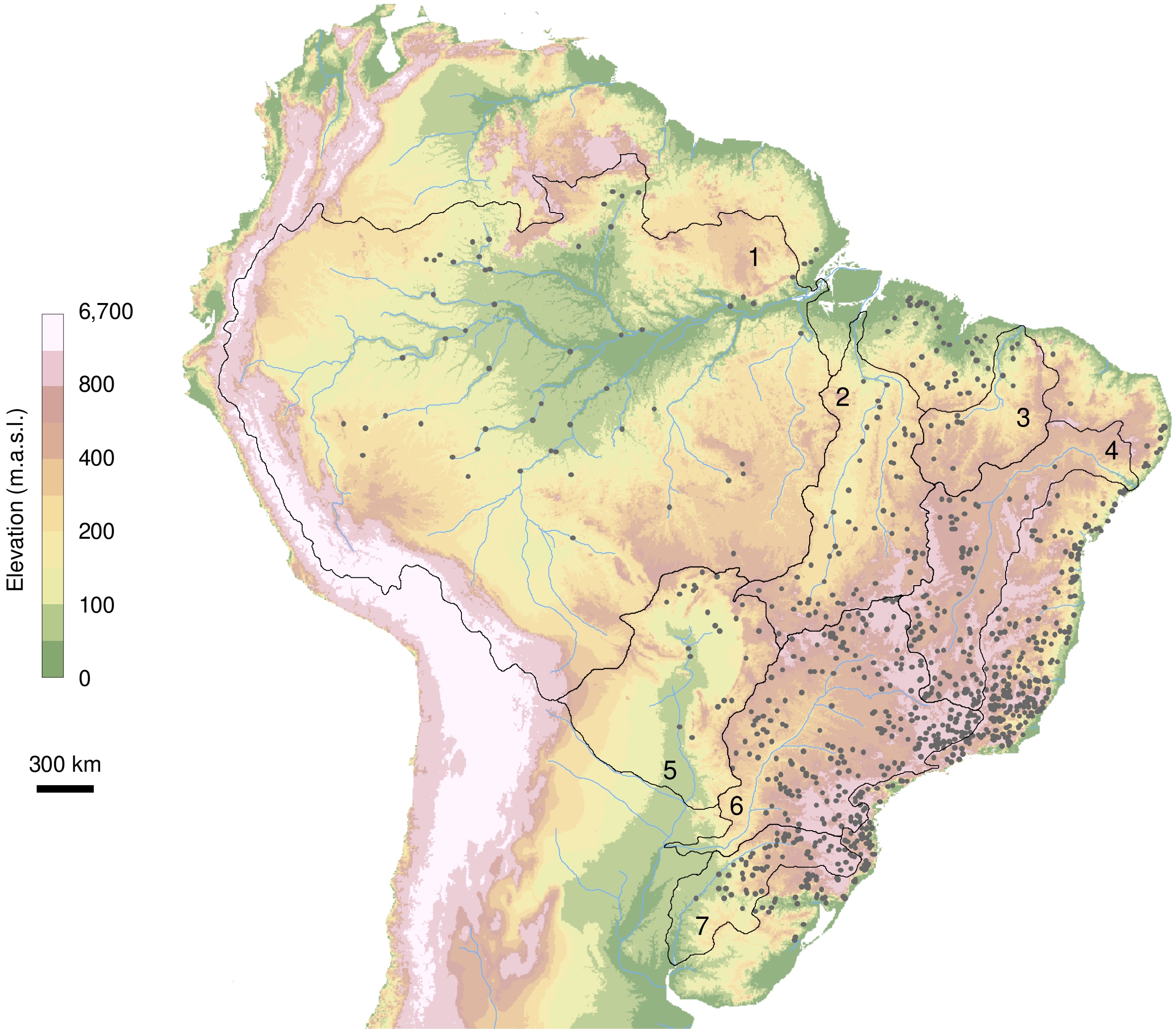

Supplementary Figure 1. **Hydrometric stations and major Brazilian river basins.** Points indicate the locations of the hydrometric stations analyzed. Black lines represent the watersheds of major Brazilian basins: 1, Amazon; 2, Tocantins-Araguaia; 3, Parnaíba; 4, São Francisco; 5, Paraguay; 6, Paraná; 7, Uruguay.

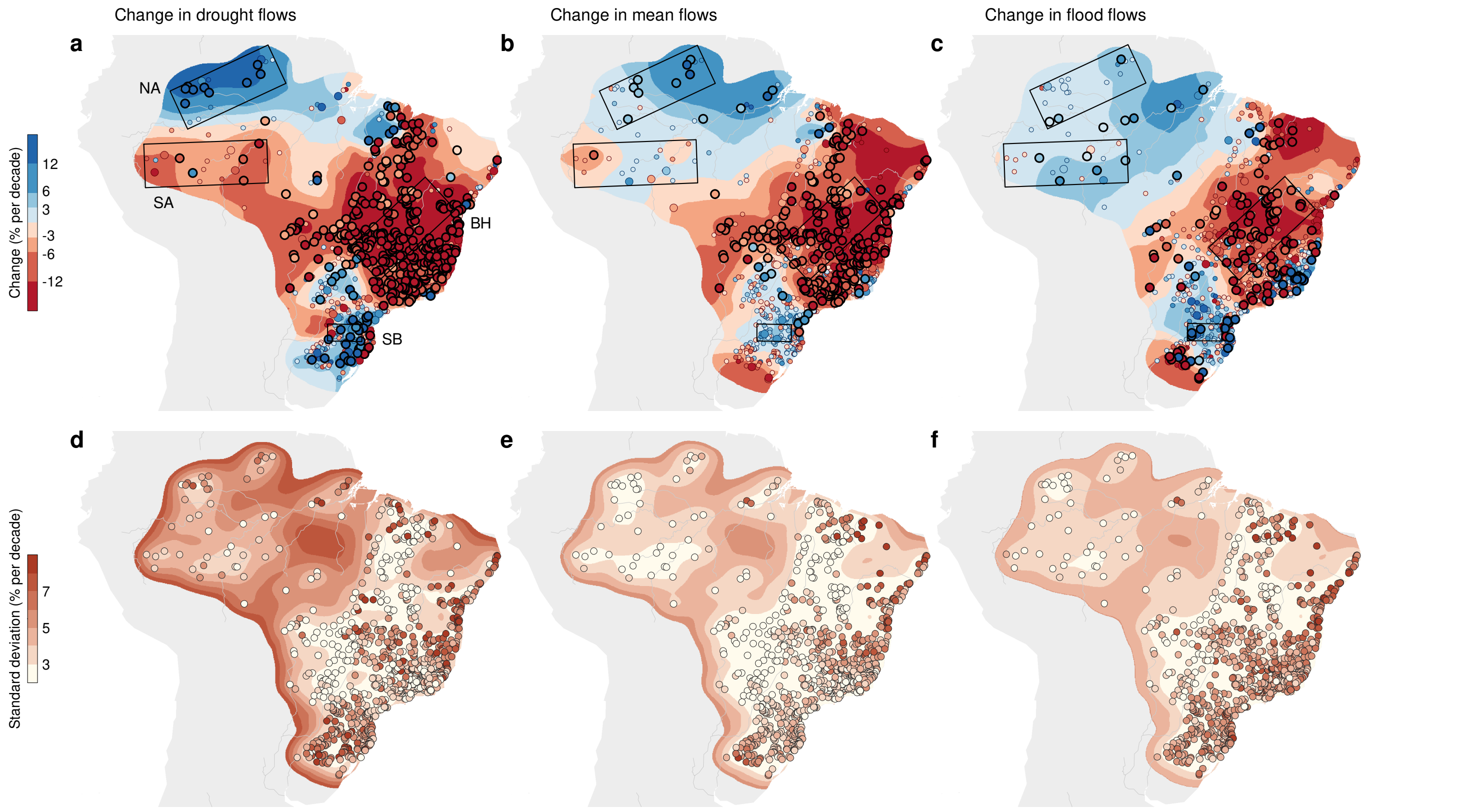

Supplementary Figure 2. **Streamflow trends in Brazil (1980-2015). a** Change in annual minimum 7-day streamflow (drought flows). **b** Change in mean daily streamflow. **c** Change in annual maximum daily streamflow (flood flows). The background pattern displays regional trends and the points display local trends (n = 886). Large circles with thick borders in **a-c** show significant trends for a significance level $\alpha = 0.10$, large circles with thin borders show significant trends for a significance level $\alpha = 0.05$, and small circles show non-significant trends. **d-f** Uncertainties of local trends (points) and regional trends obtained by kriging (background). Black rectangles indicate four hotspots of change: Northern Amazonia (NA); Southern Amazonia (SA); Brazilian Highlands (BH); and Southern Brazil (SB).

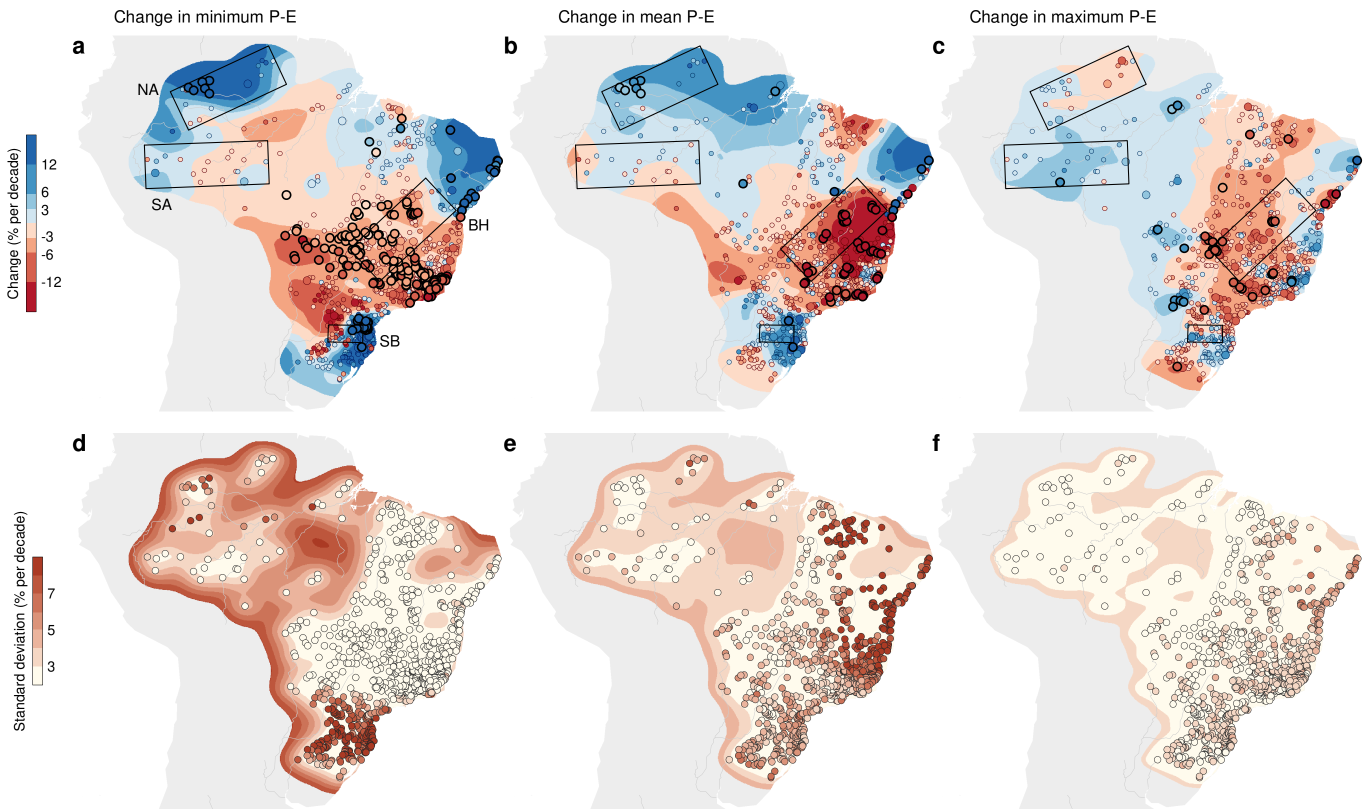

Supplementary Figure 3. **Trends of climate drivers of streamflow changes in Brazil (1980-2015). a** Change in annual minimum 90-day precipitation minus evaporation (P – E). **b** Change in mean daily P – E. **c** Change in annual maximum 14-day P – E. The background pattern displays regional trends (interpolated from local trends) and the points display local trends (which includes the contributing basin area). Large circles with thick borders in **a-c** show significant trends for a significance level α = 0.10, large circles with thin borders show significant trends for a significance level α = 0.05, and small circles show non-significant trends. **d-f** Uncertainties of local trends (points) and regional trends obtained by kriging (background). Black rectangles indicate four hotspots of change: Northern Amazonia (NA); Southern Amazonia (SA); Brazilian Highlands (BH); and Southern Brazil (SB).

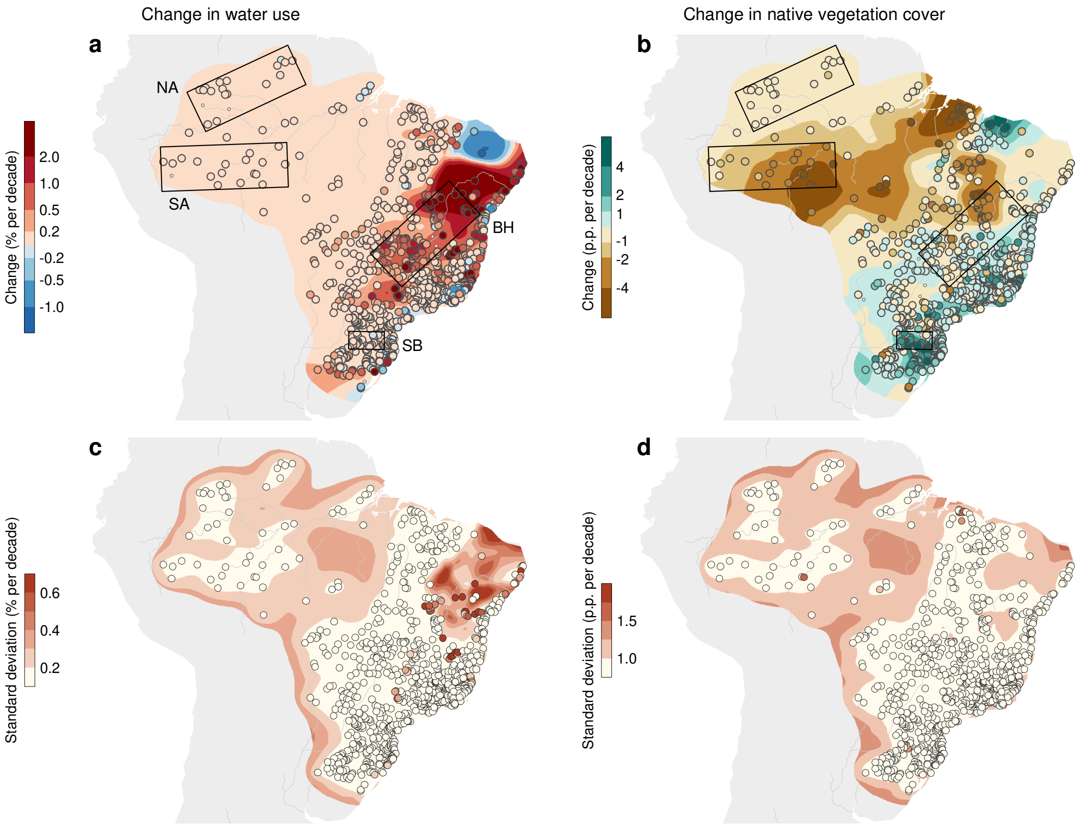

Supplementary Figure 4. **Trends of land management drivers (water use and native vegetation cover) of streamflow changes in Brazil (1980-2015). a** Change in water use in % of the long-term mean daily flow per decade. **b** Change in native vegetation cover in percentage points (p.p.) of basin area per decade. The background pattern displays regional trends (interpolated from local trends) and the points display local trends (which includes the contributing basin area). Large circles with thick borders in **a-b** show significant trends for a significance level α = 0.10, large circles with thin borders show significant trends for a significance level α = 0.05, and small circles show non-significant trends. **c-d** Uncertainties of local trends (points) and regional trends obtained by kriging (background). Black rectangles indicate four hotspots of change: Northern Amazonia (NA); Southern Amazonia (SA); Brazilian Highlands (BH); and Southern Brazil (SB).

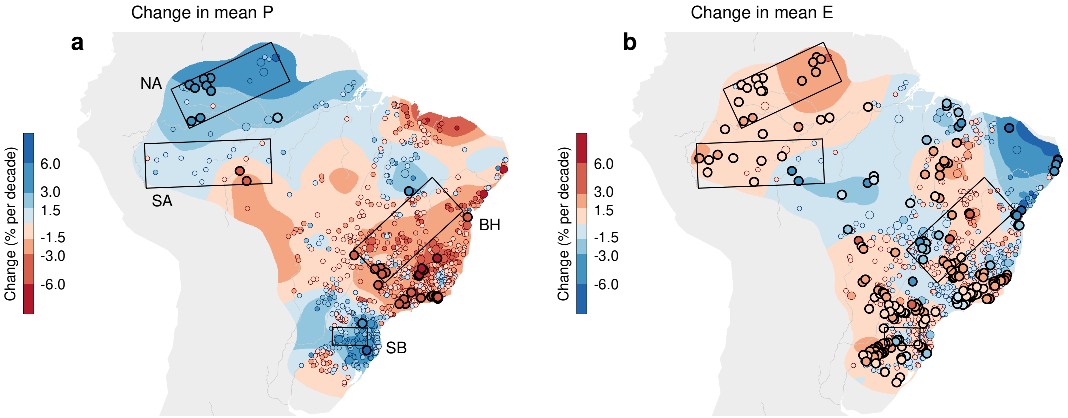

Supplementary Figure 5. **Precipitation and evaporation changes in Brazil (1980-2015). a** Change in mean daily precipitation (P). **b** Change in mean daily evaporation (E). The background pattern displays regional trends (interpolated from local trends) and the points display local trends (which includes the contributing basin area). Large circles with thick borders in show significant trends for a significance level α = 0.10, large circles with thin borders show significant trends for a significance level α = 0.05, and small circles show non-significant trends. Black rectangles indicate four hotspots of change: Northern Amazonia (NA); Southern Amazonia (SA); Brazilian Highlands (BH); and Southern Brazil (SB).

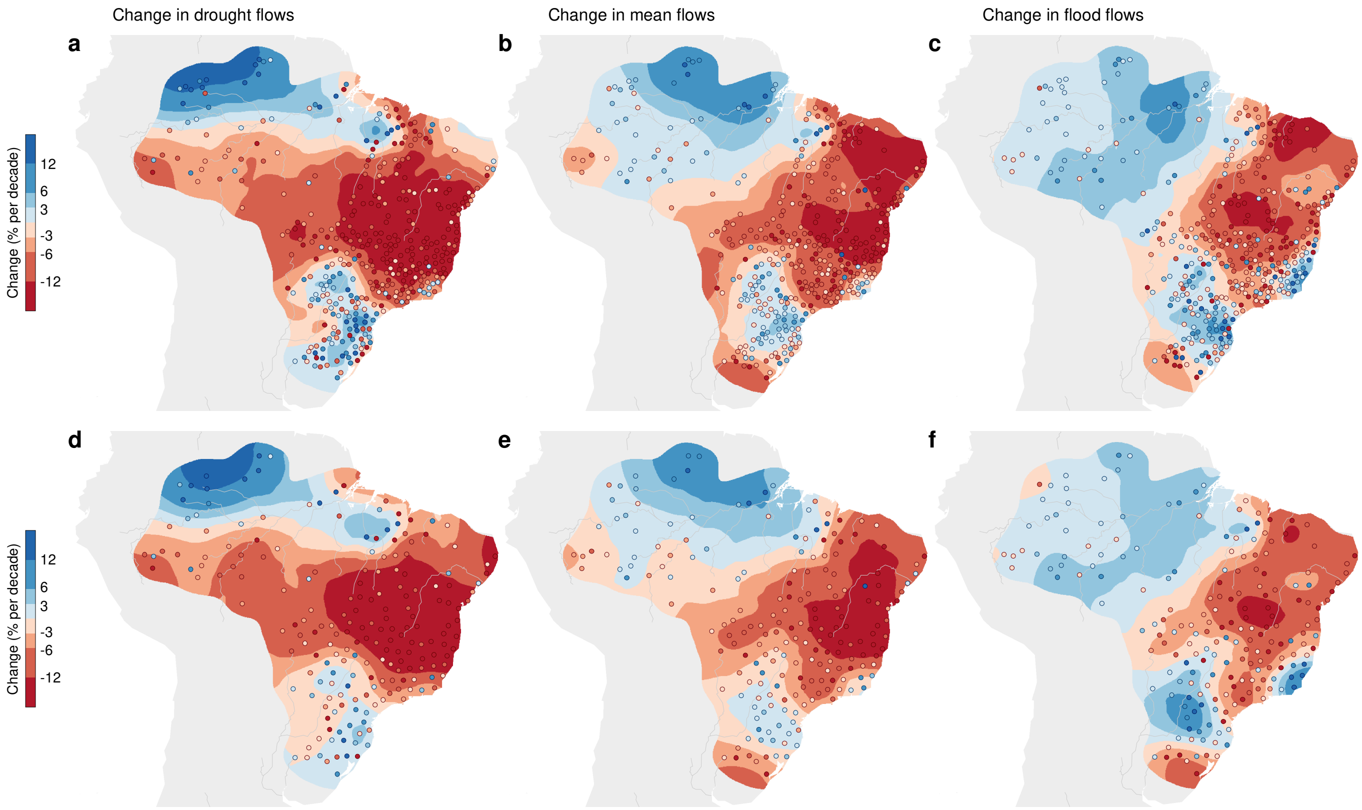

Supplementary Figure 6. **Streamflow trends as in Fig. 1 and Supplementary Fig. 2 but with fewer stations. a-c** Change in drought flows, mean flows, and flood flows using only stations with distances of at least 0.5° between each other (n = 388). **d-f** Change in drought flows, mean flows, and flood flows using only stations with distances of at least 1° between each other (n = 200).

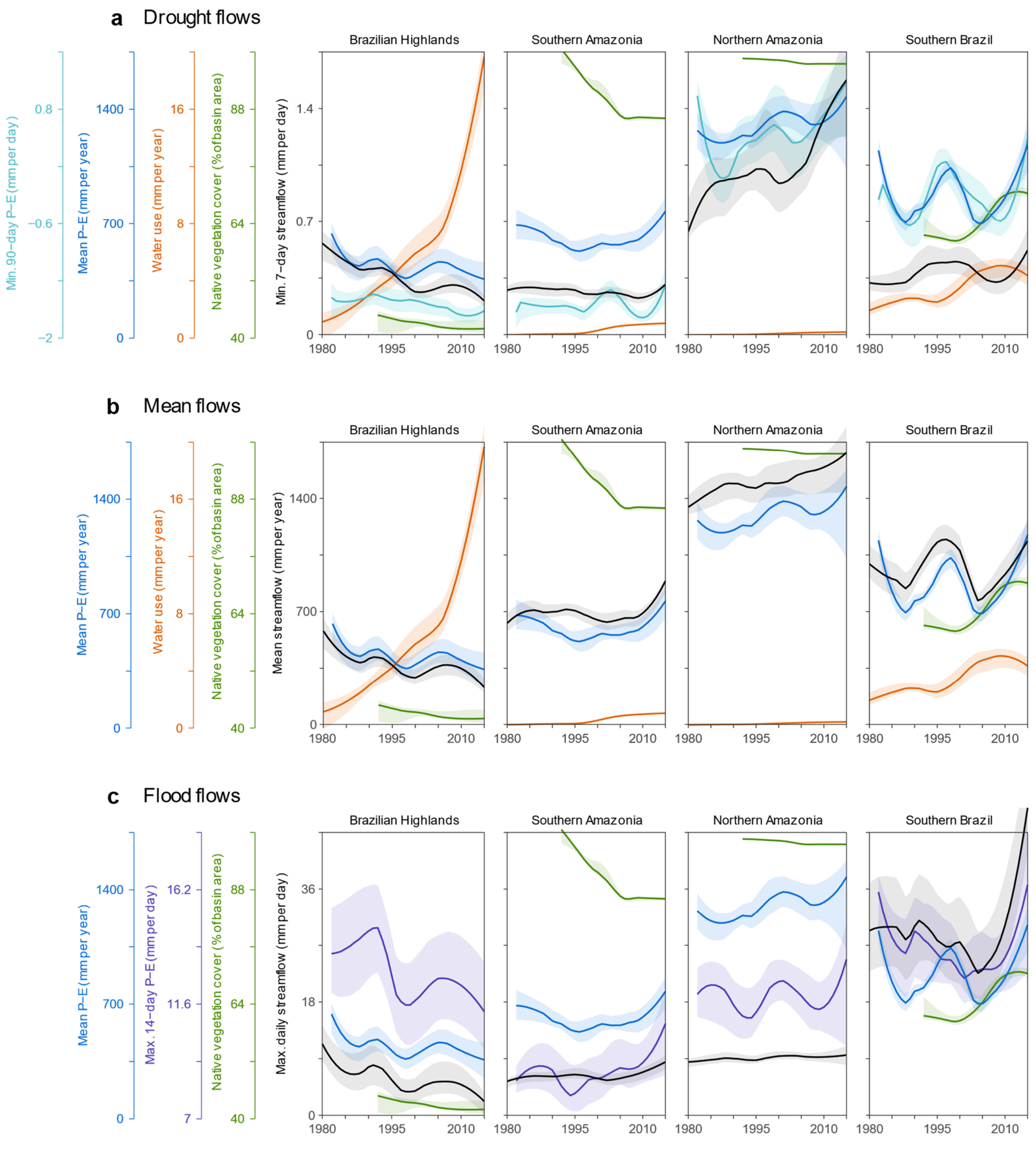

Supplementary Figure 7. **Temporal evolution of streamflow and their drivers in four hotspots of change in Brazil. a** Minimum 7-day streamflow (drought flows). **b** Mean daily streamflow (mean flows). **c** Annual maximum daily streamflow (flood flows). Black lines indicate drought flows in the upper panels, mean flows in the middle panels, and flood flows in the bottom panels. Light blue lines indicate minimum 90-day precipitation minus evaporation (P – E). Medium blue lines indicate mean P – E. Purple lines indicate maximum 14-day P – E. Orange lines indicate water use. Green lines indicate native vegetation cover. Lines represent the median and bands indicate the spatial variability within each hotspot (25th and 75th percentiles). The time series were smoothed with LOESS. The boxes represent the hotspots: Brazilian Highlands (*n* = 108); Southern Amazonia (*n* = 18); Northern Amazonia (*n* = 19); and Southern Brazil (*n* = 32).

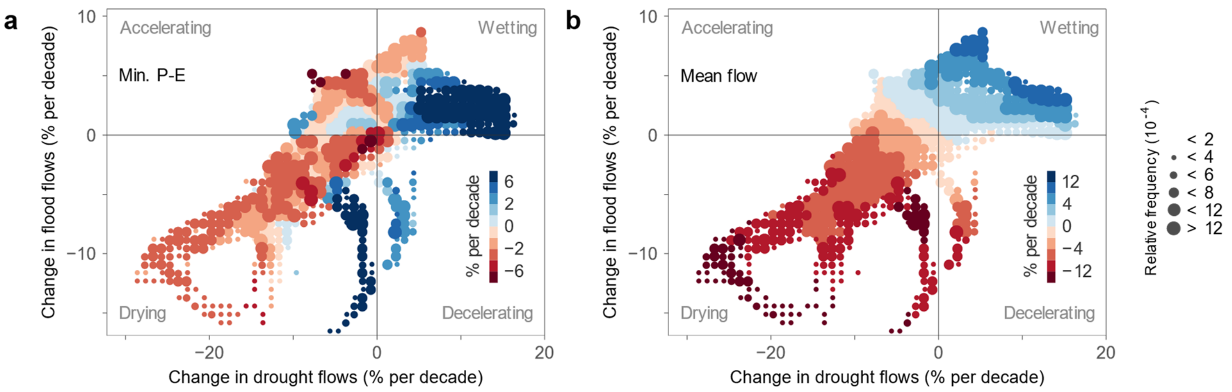

Supplementary Figure 8. **Trend of water availability and mean flows mapped on the quadrants of the accelerating, decelerating, wetting, and drying streamflow trends. a** Annual minimum 90-day precipitation minus evaporation (P – E). **b** Mean daily streamflow. Larger circles indicate higher areal fractions. Colors indicate the average trends of the drivers for each bin.

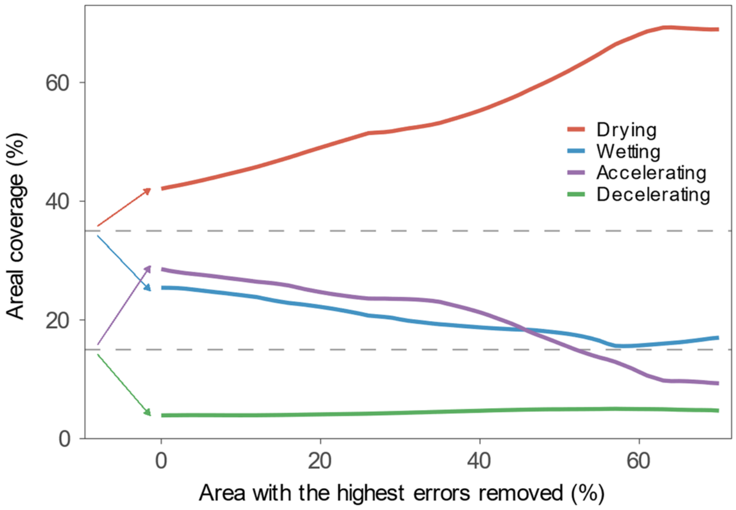

Supplementary Figure 9. **Areal coverage of each quadrant as areas with the highest errors in the regional trend estimates (kriging errors) are removed.** The dashed lines represent 15% (the expected area for the accelerating and decelerating quadrants for a bivariate distribution with ρ = 0.61 according to Equation (5) in the Supplementary Material) and 35% (the expected area for the wetting and drying trends). The arrows indicate differences from the expected values and the quadrant areal coverage when all areas are included in the quadrant classification.

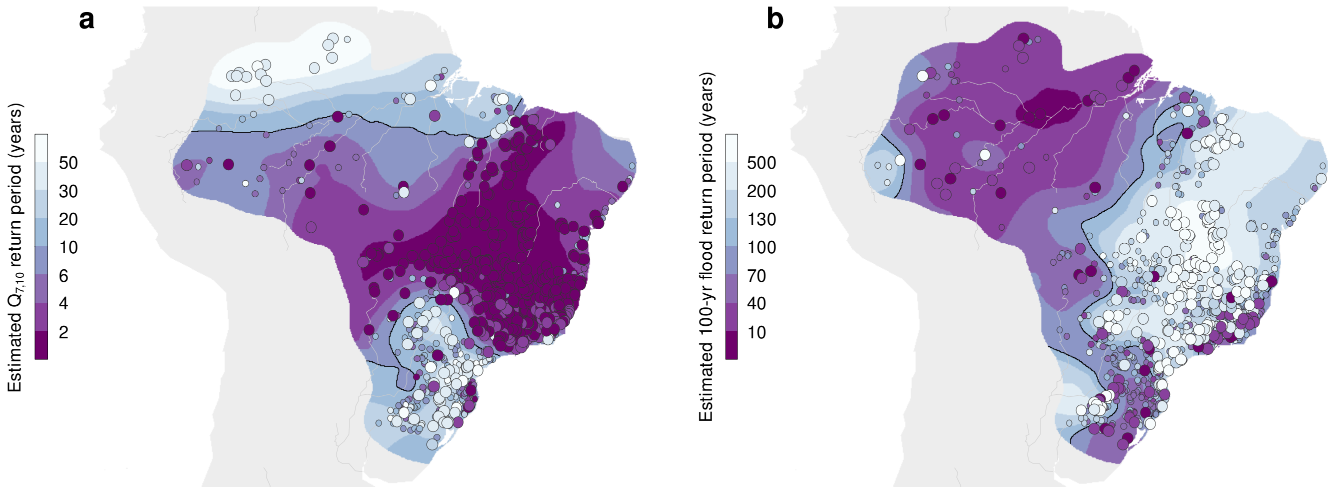

Supplementary Figure 10. **Estimated return period in 2015 for the 1980 drought and flood flows. a** Return period in 2015 for the 1980 10-year annual minimum 7-day flow ($Q_{7,10}$, drought flows). **b** Return period in 2015 for the 1980 100-year annual maximum daily streamflow (flood flows). Dark colors indicate lower return periods, representing increasing drought and flood risk. Light colors indicate higher return periods, representing decreasing risk. Points show local return periods (n = 886). Larger points indicate agreement of the 5th and the 95th percentiles of the uncertainty distribution in the sign of change. The black lines separate regions of increasing and decreasing risks.

(a)

Variable	CHIRPS	MSWEP	PERSIANN	CPC
Mean P	0.62	0.67	0.29	0.08
Maximum P	0.61	0.71	0.22	0.61
Minimum P	0.49	0.48	-0.15	0.30

(b)

Variable	CHIRPS	MSWEP	PERSIANN	CPC	ANA
Mean P	-0.09	1.92	2.61	-5.41	-1.26
Maximum P	-0.13	1.95	2.55	-3.32	-0.51
Minimum P	-4.44	-2.78	-7.06	-5.59	-2.32

Supplementary Table 1. **Comparison of precipitation trends among datasets. a** Spearman correlation coefficients between trends of the weather station series from the ANA dataset and trends in CHIRPS, MSWEP, PERSIANN and CPC datasets for: mean daily precipitation (Mean P); 14-day maximum precipitation (Maximum P); and 90-day minimum precipitation (Minimum P). **b** The median trend in each dataset for the same precipitation variables as in (A), in % per decade.
